# Pharmacological modulation of stress granules *via* G3BP1/2: A pathway to treat cancer, inflammatory disease, and neurodegeneration

**DOI:** 10.3389/fphar.2026.1780146

**Published:** 2026-03-17

**Authors:** Jinhua Yang, Fenfei Gao

**Affiliations:** Department of Pharmacology, Shantou University Medical College, Shantou, China

**Keywords:** agonists and modulators, cancer, drug development, druggable sites, G3BP1/2, inflammatory disease, neurodegeneration, stress granules (SGs)

## Abstract

Stress granules (SGs) are membraneless ribonucleoprotein condensates formed by liquid–liquid phase separation of non-translating mRNAs under stress, acting as dynamic platforms for translational reprogramming and cytoprotection. Ras-GAP SH3 domain-binding proteins 1 and 2 (G3BP1/2) are core nucleators of mammalian SGs–their dual knockout almost abolishes SG assembly, while G3BP1 overexpression alone can drive SG assembly. By sensing cytosolic RNA, G3BP1/2 couple the cyclic GMP–AMP synthase (cGAS)–STING innate immune pathway to stress signaling in cancer and neurodegeneration, positioning these proteins as central hubs linking stress-responsive translation control to disease phenotypes. Recent years have witnessed growing interest in targeting the G3BP–SG axis pharmacologically. Small molecules and peptides that bind G3BP1/2 have revealed that manipulating SG assembly/disassembly is feasible and can modulate downstream stress pathways. However, existing reviews have primarily covered G3BP structure, signaling, and pathology, without a unified focus on direct pharmacological modulators. Here, we present a comprehensive review of G3BP1/2 as druggable stress granule hubs, summarizing all currently reported direct inhibitors and activators, comparing their mechanisms, selectivity and limitations, and discussing translational opportunities and challenges across cancer, viral infection, and neurodegenerative disease contexts. By integrating these findings, we aim to provide an up-to-date framework that not only highlights the novelty of recent G3BP-directed modulators but also addresses prior reviewer concerns regarding overlap with existing literature–emphasizing how our synthesis uniquely compiles both SG inhibitors and “agonists” in one analysis. Ultimately, leveraging the G3BP1/2–SG axis may enable multi-pathway reprogramming of stress responses for therapeutic benefit.

## 1 Introduction

In recent years, membraneless organelles (MLOs), particularly SGs, have been recognized as critical regulatory nodes linking environmental stress to the reprogramming of gene expression (Jeon et al., 2025; Chin Sang et al., 2025; Pei et al., 2025; Ryu et al., 2024; Glauninger et al., 2022; Smith and Bartel, 2026). SGs are dynamic condensates that form *via* liquid–liquid phase separation of non-translating mRNAs together with multiple RNA-binding proteins when translation is stalled. By suppressing translation initiation, reorganizing mRNA fate, and interacting with other organelles, SGs help to mitigate stress-induced cellular damage (Li Y. et al., 2024; Jeon et al., 2021; Wu et al., 2025; Singh et al., 2024; Helton et al., 2025). Under physiological conditions, SGs promote cell survival under oxidative stress, hypoxia, nutrient deprivation, viral infection, and other insults. By contrast, under chronic or dysregulated conditions, aberrant SG composition and dynamics are closely linked to a range of pathological processes, including malignant tumors, neurodegenerative diseases, infections, and cardiovascular disorders (Wu et al., 2025; Zhou et al., 2023; Yuan et al., 2025; Cui et al., 2024). In cancer, SGs are increasingly recognized as adaptive hubs that help tumor cells survive intrinsic and therapy-induced stresses (e.g., hypoxia, oxidative stress, nutrient limitation, and genotoxic stress) by reprogramming translation and mRNA fate, thereby promoting stress tolerance, metabolic plasticity, and, in some settings, chemoresistance and invasive potential. Aberrant SG assembly or persistence can shift the balance from transient stress protection to sustained pro-survival signaling, linking SG dynamics to malignant progression and treatment failure (Jia et al., 2024; Asadi et al., 2021; Santofimia-Castaño et al., 2024). In inflammatory disease and innate immunity, SGs intersect with nucleic-acid sensing and antiviral defense pathways (Paget et al., 2023). SG components can modulate the availability and signaling outputs of key innate immune factors and cytokine-related mRNAs, thereby shaping type I interferon responses and inflammatory transcriptional programs (Chathuranga et al., 2023). Conversely, many pathogens actively perturb SG assembly/disassembly to favor replication, highlighting SGs as regulatory nodes at the host–pathogen interface and in immune homeostasis (Long et al., 2024). In neurodegeneration, defects in SG resolution and clearance are closely associated with neuronal vulnerability (Cui et al., 2024). Persistent SGs and stress-induced ribonucleoprotein condensates may facilitate the pathological accumulation or mislocalization of aggregation-prone RNA-binding proteins, and impaired proteostasis/autophagy can further exacerbate SG persistence, creating a feed-forward loop that contributes to synaptic dysfunction and neurodegenerative pathology (Pareek et al., 2025; Gwon et al., 2021; Nóbrega-Martins et al., 2025). Members of the Ras-GAP SH3 domain–binding protein family, G3BP1 and G3BP2, are core nucleators of mammalian SGs: simultaneous deletion of both genes almost completely abolishes SG formation under diverse stress conditions, whereas overexpression of G3BP1 alone is sufficient to drive SG assembly (Maruri-López et al., 2021; Ullmer and Semler, 2016). Structural and protein–protein interaction studies indicate that G3BP1/2 rely on multivalent interactions mediated by their nuclear transport factor 2 (NTF2)-like (NTF2L) domain, RNA recognition motif (RRM), and intrinsically disordered regions to integrate mRNAs with core SG proteins such as cell cycle–associated protein 1 (Caprin1) and ubiquitin specific peptidase 10 (USP10), thereby functioning as scaffolds and central hubs within stress-responsive post-transcriptional regulatory networks (Matsuki et al., 2013; Kang et al., 2021). In disease settings, G3BP1 is overexpressed in multiple cancers (such as colorectal cancer) and is associated with activation of the Ras/ERK pathway, enhanced cell proliferation, and chemoresistance. In addition, G3BP1/2 participate in cGAS–STING–mediated DNA sensing and in the functional coupling of SGs with lysosomes, autophagy-related compartments, and other membraneless organelles, thereby exerting major effects on tumor immunity, viral replication, and neuronal vulnerability (Zhou et al., 2023; Yuan et al., 2025; Legrand et al., 2020; Zhao et al., 2022). Collectively, these findings suggest that targeting the G3BP1/2–SG axis may enable “multi-target reprogramming” of translational stress responses and multiple pathogenic signaling pathways, thereby providing a solid biological rationale and structural starting point for the development of novel small-molecule inhibitors, peptide-based interventions, and natural-product agonists (Freibaum et al., 2024; Li J. et al., 2024; Fang et al., 2025).

Existing reviews on G3BP1/2 mainly cover structure, signaling, and disease links (Guo et al., 2024; Hofmann et al., 2021; Baymiller and Moon, 2023; Millar et al., 2023; Garg et al., 2025; Reineke and Lloyd, 2013; Jayabalan et al., 2023; Rajendira et al., 2026; Zhao and Li, 2025). Kang et al. (2021) summarized domain architecture, interaction networks, and antiviral/innate-immune functions. Ge et al. (2022) reviewed G3BP1 across cancers and non-cancer diseases and compiled reported modulators. Mukhopadhyay and Zhou (2023) highlighted G3BPs as nodal regulators with pharmacological potential. However, molecular-level pharmacological modulation remains insufficiently synthesized, with no systematic medicinal-chemistry overview of direct inhibitors/activators.

**FIGURE 1:**
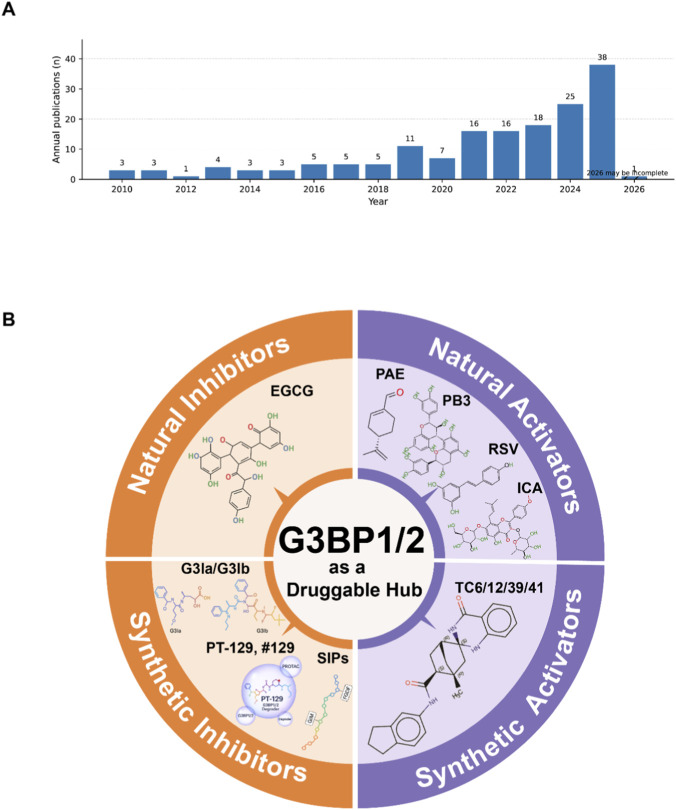
Publication trend and chemical landscape of reported G3BP1/2 modulators. **(A)** Annual number of publications reporting G3BP1/2-targeting or G3BP1/2-associated chemical modulators. Numbers above bars indicate the publication count per year; data for 2026 may be incomplete due to partial-year indexing. **(B)** Schematic overview of G3BP1/2 as a druggable hub, summarizing representative classes of modulators: natural inhibitors (e.g., EGCG), synthetic inhibitors (e.g., G3Ia/G3Ib, PT-129/#129, and stress-granule inhibitory peptides, SIPs), natural activators (e.g., PAE, PB3, RSV, ICA), and synthetic activators (e.g., TC6/12/39/41). Chemical structures are shown as illustrative examples and are not to scale.

We retrieved 147 publications related to pharmacological modulation of the G3BP1/2-centered stress granule (SG) axis (Figure 1A). The annual output has increased steadily, indicating growing interest in targeting G3BP1/2 and SG assembly/LLPS. This expanding literature supports the need for a systematic synthesis to define actionable binding sites, distinguish inhibitor-versus-agonist modalities, and provide a framework for developing the G3BP-driven SG pathway as a druggable target.

This review builds on prior structural and disease-focused overviews. We highlight the structural basis that makes G3BP1/2 “druggable” stress hubs, summarize reported direct inhibitors and agonists (Figure 1B), and assess translational opportunities and key challenges across cancer therapy, antiviral strategies, and neuroprotection. In doing so, we aim to provide an integrated conceptual framework to inform the rational optimization and preclinical evaluation of future G3BP-targeted modulators. Next, we map G3BP1/2 domain architecture, key PTMs, and validated interfaces that define druggable sites.

## 2 Structural basis and druggable sites of G3BP1/2

We first outline domain architecture, key PTMs, and validated druggable sites that enable pharmacological control of G3BP1/2.

### 2.1 Domain architecture and intrinsically disordered regions

**FIGURE 2:**
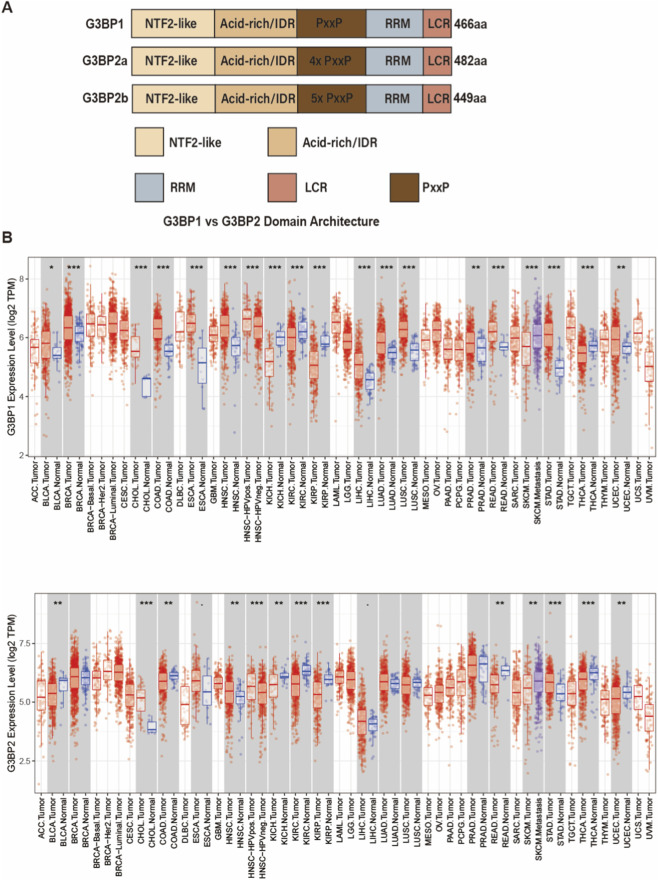
Domain architecture and pan-cancer expression of G3BP1 and G3BP2. **(A)** Schematic domain organization of human G3BP1, G3BP2a and G3BP2b, highlighting the NTF2L domain, acid-rich/intrinsically disordered region (IDR), multiple proline-rich PxxP motifs, RNA-recognition motif (RRM) and low-complexity region (LCR), with total amino-acid length indicated to the right. **(B)** Pan-cancer comparison of G3BP1 (top) and G3BP2 (bottom) mRNA levels across TCGA/GTEx cohorts. Boxplots show log_2_(TPM) expression in tumors (red) *versus* matched or GTEx normal tissues (blue), depending on normal-sample availability for each cancer type; grey shading separates tumor–normal pairs. *P* values were calculated using a two-sided Wilcoxon rank-sum test (tumor vs. normal). Asterisks denote statistical significance for tumor *versus* normal comparisons (**p < 0.01; ***p < 0.001; ns, not significant). Cancer type abbreviations: ACC, Adrenocortical carcinoma; BLCA, Bladder urothelial carcinoma; BRCA, Breast invasive carcinoma; BRCA–Basal, Breast invasive carcinoma (Basal-like); BRCA–Her2, Breast invasive carcinoma (HER2-enriched); BRCA–Luminal, Breast invasive carcinoma (Luminal); CESC, Cervical squamous cell carcinoma and endocervical adenocarcinoma; CHOL, Cholangiocarcinoma; COAD, Colon adenocarcinoma; COADREAD, Colorectal adenocarcinoma; DLBC, Diffuse large B-cell lymphoma; ESCA, Esophageal carcinoma; GBM, Glioblastoma multiforme; HNSC, Head and neck squamous cell carcinoma; HNSC–HPVneg, Head and neck squamous cell carcinoma (HPV-negative); HNSC–HPVpos, Head and neck squamous cell carcinoma (HPV-positive); KICH, Kidney chromophobe; KIRC, Kidney renal clear cell carcinoma; KIRP, Kidney renal papillary cell carcinoma; LAML, Acute myeloid leukemia; LGG, Lower grade glioma; LIHC, Liver hepatocellular carcinoma; LUAD, Lung adenocarcinoma; LUSC, Lung squamous cell carcinoma; MESO, Mesothelioma; OV, Ovarian serous cystadenocarcinoma; PAAD, Pancreatic adenocarcinoma; PCPG, Pheochromocytoma and paraganglioma; PRAD, Prostate adenocarcinoma; READ, Rectum adenocarcinoma; SARC, Sarcoma; SKCM, Skin cutaneous melanoma; SKCM–Metastasis, Skin cutaneous melanoma (metastatic); SKCM–Tumor, Skin cutaneous melanoma (primary tumor); STAD, Stomach adenocarcinoma; TGCT, Testicular germ cell tumor; THCA, Thyroid carcinoma; THYM, Thymoma; UCEC, Uterine corpus endometrial carcinoma; UCS, Uterine carcinosarcoma; UVM, Uveal melanoma.

G3BP1 and G3BP2 are highly conserved in overall architecture, each comprising an N-terminal NTF2L domain, an adjacent acid-rich/intrinsically disordered region (acid-rich/IDR), a central proline–X–X–proline (PXXP)–enriched segment, and C-terminal RRM and RGG/low-complexity (RGG/LCR) regions (Parker et al., 1996; Kennedy et al., 2001; Kedersha et al., 2016; Panas et al., 2015). However, they differ markedly in the number of PxxP motifs: G3BP1 contains only a single PxxP motif, whereas G3BP2a and G3BP2b contain four and five PxxP motifs, respectively, suggesting that the latter exhibit higher valency for binding SH3-domain proteins and signaling complexes and may therefore possess stronger scaffold and signal-integration capacities (Figure 2A) (Kang et al., 2021; Sidibé et al., 2021; He et al., 2021; Vognsen et al., 2013).

Crystallographic and cryo-electron microscopy studies have shown that the NTF2L domain forms a conserved hydrophobic groove framed by α-helices and β-strands, which specifically recognizes a variety of protein ligands containing phenylalanine–glycine–aspartate–phenylalanine (FGDF) motifs, including alphaviral non-structural protein 3 (nsP3), the SARS-CoV-2 N protein, and SG regulators such as Caprin1 and USP10; these stress granule–associated proteins engage G3BP1/2 by binding to this pocket to form stable complexes (Long et al., 2024; Zheng et al., 2019; Jin et al., 2022; Schulte et al., 2016). The same pocket also serves as the binding site for the recently described small-molecule inhibitors G3Ia and G3Ib, which competitively occupy the NTF2L hydrophobic groove, thereby blocking the interaction of G3BP1/2 with ligands such as Caprin1 and ultimately suppressing or dissolving stress granules (Freibaum et al., 2024). By contrast, the C-terminal IDR, RRM, and RGG regions are mainly responsible for recognizing mRNAs and various RNA-binding proteins and for mediating multivalent associations, constituting the core driving module for G3BP1/2 liquid–liquid phase separation (LLPS): deletion of specific IDR segments markedly impairs mRNA recruitment, and mature condensates cannot form even when the RRM/RGG regions are preserved (He et al., 2021; Guillén-Boixet et al., 2020; Yang et al., 2020).

Yang et al. (2020), together with Guillén-Boixet et al. (2020), used *in vitro* reconstitution and live-cell imaging to propose that G3BP1 exists in an RNA-dependent equilibrium between autoinhibited and active states. Under low-RNA conditions, intramolecular association between the NTF2L domain and the acidic IDR maintains a closed conformation and suppresses LLPS. When cytosolic free RNA rises sharply under stress, unfolded mRNAs competitively disrupt this autoinhibitory interface, promoting G3BP1 clustering and LLPS together with RNA and other core SG proteins, thereby nucleating stress granule assembly.

### 2.2 Post-translational modifications and functional regulation

G3BP1/2 undergo extensive post-translational modifications under diverse stress conditions, including serine phosphorylation, lysine ubiquitination, and arginine methylation/demethylation within the RGG region. These modifications finely tune their LLPS capacity and the dynamics of SG assembly and disassembly (Yuan et al., 2025; Sidibé et al., 2021). Multiple studies indicate that the phosphorylation status of Ser149 and related residues regulates the conformational “open–closed” state of G3BP1 by modulating intramolecular interactions between IDRs: dephosphorylation at S149 generally favors G3BP1 dimerization and SG assembly, whereas re-phosphorylation promotes SG disassembly upon stress resolution (Barr et al., 2013). Under heat shock, Gwon et al. (2021) found that G3BP1 undergoes K48- and K63-linked ubiquitination and forms a disassembly complex with the ER-associated protein FAF2 and the ATPase valosin-containing protein (VCP; also known as p97). This complex selectively extracts ubiquitinated G3BP1 from SGs, constituting a key step in SG dissolution following thermal stress. In addition, Tsai et al. (2016), Tsai et al. (2017) along with subsequent studies, showed that asymmetric arginine methylation within the G3BP1 RGG region suppresses the formation of large SGs, whereas rapid demethylation mediated by jumonji domain-containing protein 6 (JMJD6) under stresses such as arsenite acts as a molecular switch that promotes SG assembly. Knockdown of JMJD6 or perturbation of the methylation status of G3BP1 markedly alters both the size and number of SGs (Kang et al., 2021).

### 2.3 Druggable sites derived from structure and post-translational modifications

#### 2.3.1 NTF2L pocket

The conserved hydrophobic groove within the NTF2L domain of G3BP1/2 is not only a binding hotspot for FGDF motif–containing proteins such as nsP3, the SARS-CoV-2 N protein, and Caprin1/USP10, but also the principal binding site for all currently reported direct small-molecule inhibitors (G3Ia/G3Ib) and multiple NTF2L-targeting peptides. It is therefore regarded as the most firmly validated and, to date, the most extensively explored druggable pocket on G3BP1/2 (Freibaum et al., 2024; Song et al., 2022). Importantly, however, simple “occupancy” of the NTF2L pocket does not uniquely predict phenotypic outcome: ligands reported to bind this groove can either promote SG assembly (apparent agonism), consistent with recent efforts to engineer NTF2L as a small-molecule agonist site that enhances G3BP1 phase-separation capacity, or suppress SG assembly and/or accelerate SG dissolution (apparent antagonism). Collectively, these findings indicate that the NTF2L pocket is not a unidirectional inhibitory site but a bidirectional control point whose functional output depends on how ligand engagement reshapes partner competition and the underlying condensation network (Vognsen et al., 2013; Kristensen, 2015).

#### 2.3.2 C-terminal RNA-binding interface and IDR regulatory region

The C-terminal RRM/arginine–glycine–glycine (RGG)–rich region and its adjacent IDRs drive G3BP1/2 LLPS through multivalent RNA binding and protein–protein interactions, and constitute a key interface that determines SG assembly capacity and mRNA selectivity (Yang et al., 2020) (Huang et al., 2021; Lavrynenko et al., 2025). In principle, ligands that block RNA binding or alter IDR conformation could be designed to finely tune the phase-separation threshold of G3BP1/2 and reshape the spectrum of client RNAs that they recruit. However, current chemical tools are largely confined to the NTF2L pocket, and there are still very few reports of small molecules or peptides that directly target the RRM/RGG–IDR axis to modulate G3BP1/2 LLPS. Most pharmacological exploration of this region remains at the conceptual or structure–function level, highlighting it as a promising yet underexplored druggable space for future investigation (Van Nerom et al., 2024; Götte et al., 2019).

#### 2.3.3 Ubiquitination–degradation interface and proteolysis-targeting chimera (PROTAC) design

Heat shock–induced ubiquitination of G3BP1 and its engagement with the p97/FAF2-dependent protein clearance pathway indicate that G3BP1/2 are intrinsically connected to the intracellular protein quality-control and degradation machinery, providing a biological rationale for harnessing the ubiquitin–proteasome system to selectively eliminate G3BP1/2 (Gwon et al., 2021) (Tolay and Buchberger, 2022; Krause et al., 2022; Maxwell et al., 2021; Hou and Tang, 2023; Müller, 2021). By coupling an NTF2L domain ligand (G3BP1/2-targeting ligand-1) to an E3 ligase–recruiting “molecular glue”, Dong et al. (2024) developed the first G3BP1/2-directed PROTAC degrader, PT-129, which efficiently degrades G3BP1/2 in multiple cell types, suppresses SG biogenesis and dissolves pre-existing SGs, thereby blocking activating transcription factor 4 (ATF4)-dependent migracytosis and associated tumor growth. This work validates the “ubiquitination–degradation interface” as a feasible druggable site on G3BP1/2.

## 3 The roles of the G3BP1/2–stress granule axis in cancer, inflammatory disease, and neurodegeneration

Having defined druggable sites, we next link the G3BP1/2–SG axis to disease mechanisms that motivate therapeutic modulation.

### 3.1 Cancer

Pan-cancer analyses based on The Cancer Genome Atlas (TCGA) and Genotype-Tissue Expression (GTEx) datasets indicate that *G3BP1* mRNA is significantly upregulated in most solid tumors, including breast, lung adenocarcinoma, colorectal, gastric, and hepatocellular carcinomas, compared with their normal counterparts (Li et al., 2017; Li et al., 2016). Across multiple cancer types, elevated G3BP1 expression is associated with poor prognosis, altered immune-cell infiltration, and activation of several oncogenic signaling pathways (such as JAK–STAT and PI3K–AKT), suggesting that G3BP1 has potential as a diagnostic, prognostic, and immune-related biomarker (Lu et al., 2025; Zhang et al., 2021; Liu S. et al., 2022; Zheng et al., 2019; Li Y. et al., 2020; Alshaker et al., 2019) (Figure 2B).

G3BP2 is persistently overexpressed in multiple malignancies, including breast cancer, gastric cancer, non–small cell lung cancer, esophageal squamous cell carcinoma, and osteosarcoma. Its protein levels tend to increase with advancing tumor stage and are closely associated with poor prognosis, suggesting that G3BP2 may serve as a potential prognostic biomarker and therapeutic target (Zhang et al., 2015).

Mechanistically, G3BP2 stabilizes multiple oncogenic transcripts *via* its RRM domain. In breast cancer, it binds to and stabilizes squamous cell carcinoma antigen recognized by T cells 3 (*SART3*) mRNA, thereby maintaining the tumor-initiating cell state and driving tumorigenesis; in esophageal squamous cell carcinoma, it stabilizes hepatoma-derived growth factor (*HDGF*) mRNA and forms a long intergenic non-coding RNA 01554 (*LINC01554*)–G3BP2–HDGF axis that promotes invasion and metastasis (Gupta et al., 2017; Zheng et al., 2022).

Beyond its role in RNA metabolism, G3BP2 also acts as a scaffold that controls the subcellular localization of key signaling proteins. By interacting with IκBα, G3BP2 enhances nuclear accumulation of NF-κB p65 and NF-κB–dependent transcription, thereby promoting pathological cardiac hypertrophy and vascular inflammation (Prigent et al., 2000; Hong et al., 2018; Li T. et al., 2020). In prostate cancer, androgen-induced G3BP2 forms a complex with tumor protein p53 (p53) and the small ubiquitin-like modifier (SUMO) E3 ligase RAN binding protein 2 (RanBP2), with tripartite motif-containing protein 25 (TRIM25) as a co-regulator, to drive p53 SUMOylation and nuclear export, attenuating p53-mediated tumor suppression and contributing to therapy resistance (Ashikari et al., 2017; Takayama et al., 2018). In addition, in colorectal cancer, RIO kinase 1 (RIOK1)-dependent phosphorylation of G3BP2 facilitates mouse double minute 2 homolog (MDM2)-mediated ubiquitination and degradation of p53, which underlies G3BP2-associated radioresistance (Shechter et al., 2023). Tumor cells exploit G3BP1/2-nucleated SGs to survive hypoxia/oxidative stress and therapy-induced stress, and SG biology is increasingly linked to treatment resistance (Redding and Grabocka, 2023; Zhao et al., 2021). In obesity-associated pancreatic ductal adenocarcinoma models, elevated SG dependence was shown to be a determinant of tumor development, supporting SG-pathway intervention as a context-specific therapeutic vulnerability (Fonteneau et al., 2022). A G3BP1/2-targeting degrader (PT-129) has been reported to inhibit SG formation/disassemble pre-existing SGs and to produce anti-tumor effects *via* an SG-dependent ATF4 migracytosis mechanism in preclinical settings, illustrating a target-engagement-forward therapeutic strategy (Dong et al., 2024). Because degraders and peptides often face solubility and biodistribution constraints, nanocarrier approaches (“Nano-PROTACs” and related systems) are being developed to improve delivery and therapeutic index and could, in principle, be adapted for G3BP1/2 degraders such as PT-129 (Arenas-Moreira et al., 2026; Amalraj and Parthasarathy, 2026). Combination regimens are supported by evidence that G3BP1 depletion radiosensitizes lung cancer cells and that high G3BP1 correlates with poorer outcomes after adjuvant chemotherapy in gastric cancer cohorts, consistent with SG/G3BP modulation as a sensitization axis (Cho et al., 2019). Recent work suggests that nanoscale material “signatures” can reprogram the state of biomolecular condensates, including SGs, implying that nanomaterials may influence condensate biology beyond serving as delivery vehicles (Zheng et al., 2025). In addition, protein nanoparticles can perturb cellular homeostasis and trigger stress responses, in which SGs may act as a buffering/protective mechanism—highlighting potential translational confounders and safety considerations for nanomedicine-based interventions (Song et al., 2025). Building on this concept, combination nanotherapies have emerged in which anticancer agents are co-encapsulated with SG-suppressing modulators within nanoparticles to enhance efficacy and counter stress-adaptive resistance. A liposomal HY+IM nanodrug (HINPs) was reported to enhance photodynamic therapy by disrupting the G3BP1–UBAP2L complex, thereby suppressing stress granule (SG) formation (Hu X. et al., 2025). In hepatocellular carcinoma models, metal–polyphenol network–coated nanoparticles (MPN-R612F) were shown to reduce sorafenib resistance by inhibiting SG assembly (Zhou et al., 2024).

### 3.2 Inflammatory disease and innate immunity

G3BP1 and G3BP2 act as central scaffolds for stress-granule and RNA–protein condensate biology and are repeatedly discussed as intervention points that can reshape innate-immune signaling and downstream inflammatory outputs (Kang et al., 2021). In nucleic-acid–driven inflammation, G3BP1 has been shown to promote cytosolic DNA sensing by physically supporting cGAS activation, which can amplify type I interferon programs relevant to autoinflammatory disease (Liu et al., 2019). The natural product epigallocatechin gallate (EGCG) can disrupt the G3BP1–cGAS complex and inhibit DNA-triggered cGAS activation and interferon production, with reported mitigation of self-DNA–driven autoinflammation in an Aicardi–Goutières syndrome mouse model and reduced interferon-stimulated gene expression in patient cells (Liu et al., 2019). Resveratrol has also been reported to suppress both intracellular DNA- and RNA-induced type I interferon production through inhibiting G3BP1 and to alleviate nucleic-acid–stimulated autoimmune responses in experimental mouse models of Aicardi–Goutières syndrome (Cai et al., 2021).

### 3.3 Neurodegeneration and axonal regeneration

In neurodegenerative disorders, persistent or aberrantly matured SGs and related ribonucleoprotein condensates are increasingly linked to proteostasis failure and neuronal vulnerability, making the G3BP1/2-centered SG pathway a plausible therapeutic leverage point (Cui et al., 2024). In a neurodegeneration-relevant model, axonal TDP-43 drives G3BP1-positive condensates that suppress local translation, and experimentally dissociating G3BP1 condensates restores local translation and alleviates axonal toxicity, supporting G3BP1-targeted modulation as a mechanism-based neuroprotective strategy (Altman et al., 2021). Sahoo et al. (2018) proposed that axonal G3BP1 stress granule–like assemblies function as an mRNA storage depot and a translational “gate” in axons, and they developed a cell-penetrating minimal functional peptide (Tat-G3BP1, amino acids 190–208) that promotes axon outgrowth across species and multiple neuronal models. Sahoo et al. (2020) described a Ca^2+^-dependent translational switch in injured peripheral axons, in which local translation of *mTOR* mRNA is required to enable subsequent local translation of axonal Csnk2a1 mRNA (CK2α); the newly synthesized CK2α phosphorylates G3BP1 (Ser149) to trigger axonal G3BP1-granule disassembly, thereby releasing sequestered axonal mRNAs for local translation and promoting axon regrowth/nerve regeneration. van Erp et al. (2021) used a neuronal culture system that enables physical separation of axons, and combined laser axotomy with proteomics and puromycin/O-propargyl-puromycin (OPP)-based nascent protein labeling to directly link regenerative capacity to local axonal translation. They further applied a cell-penetrating dominant-negative peptide, G3BP1 (190–208), to disrupt/compete with axonal G3BP1 granules and thereby release associated mRNAs and factors; however, in mature axons, this peptide neither increased OPP-measured protein synthesis nor improved the regeneration rate, leading the authors to propose that the key bottleneck in mature axons is insufficient translational machinery/declining translational competence, rather than limited mRNA availability. In their Proceedings of the National Academy of Sciences 2025 study (published 27 February 2025), Sahoo et al. (2025) leveraged and systematically optimized a previously validated cell-penetrating peptide (CPP) spanning rat G3BP1 residues 190–208 (an acidic segment previously shown to promote axon growth in cultured neurons), and showed that the internal EP7 repeat core (residues 192–205) is sufficient for neurite/axon growth–promoting activity, whereas scrambling the alternating acidic–proline repeat pattern abolishes activity; they further extended these findings from *in vitro* systems to mammalian central nervous system (CNS) injury paradigms, including spinal cord injury with a peripheral nerve graft and an optic nerve crush model. Together, these studies highlight that pharmacological modulation of the G3BP1/2–SG axis can have compartment-specific and context-dependent outcomes, underscoring the need to consider tissue specificity, timing, and potential risks of chronic SG perturbation when translating G3BP-directed strategies to neurodegeneration and neural repair.

We then review direct pharmacological modulators, beginning with inhibitors and degraders that suppress SG assembly or accelerate SG dissolution.

## 4 Direct inhibitors that directly target G3BP1/2

For each modality, we summarize binding site, evidence for target engagement, and key cellular/*in vivo* phenotypes.

### 4.1 Synthetic inhibitors

#### 4.1.1 NTF2L pocket small molecules: G3Ia and G3Ib

On the basis of the alphaviral nsP3–G3BP1 complex and the crystal structure of the G3BP1 NTF2L domain, Freibaum et al. (2024) used structure-guided design combined with fragment optimization to generate two peptidomimetic small molecules, G3BP inhibitor a (G3Ia) and G3BP inhibitor b (G3Ib). Biophysical measurements showed that G3Ia binds G3BP1/2 with submicromolar affinity (surface plasmon resonance (SPR) and isothermal titration calorimetry (ITC) K_d ≈ 0.3–0.6 μM), whereas G3Ib displays slightly weaker but still low-micromolar affinity, and exhibits negligible binding to other NTF2L proteins, indicating good selectivity for the G3BP NTF2L pocket. In in vitro phase-separation systems, G3Ia/G3Ib disrupt the multivalent RNA–G3BP1–Caprin1 network, suppress G3BP1-driven liquid–liquid phase separation, and attenuate the Caprin1-mediated pro-SG effect, while exerting only minor effects on the USP10–G3BP1 complex. Across multiple mammalian cell types, G3Ia/G3Ib suppresses SG formation and rapidly dissolves pre-existing SGs after stress. Global translation is only modestly affected by poly(L)-lysine–based labeling, supporting a primary effect on G3BP-dependent condensates rather than broad translational inhibition. In disease-relevant models, G3Ia has been used to validate the role of G3BP1-driven SGs in proteotoxicity and inflammation. For example, in human induced pluripotent stem cell (iPSC)-derived neurons and cells carrying pathogenic VCP mutations, G3Ia is able to dissolve abnormally persistent SGs and alleviate SG burden associated with neurodegeneration. This study has several limitations. Many of the key cellular experiments required relatively high concentrations (20–50 µM) to achieve strong effects—for example, at 50 µM the compounds inhibited heat shock–induced stress granules by ∼79–82%, and the granule dissolution assays were also primarily performed at 50 µM. This creates an apparent in vitro–to–cellular gap relative to the submicromolar SPR (K_D) values, suggesting that factors such as limited cell permeability, active efflux, or competition with intracellular ligands may constrain intracellular target engagement. However, the study did not fully resolve this issue by directly measuring intracellular target occupancy or intracellular compound concentrations. Overall, the work remains largely focused on cellular and *in vitro* systems, and it is still far from drug development requirements such as PK profiling, blood–brain barrier (BBB) penetration, and efficacy in animal disease models.

**FIGURE 3:**
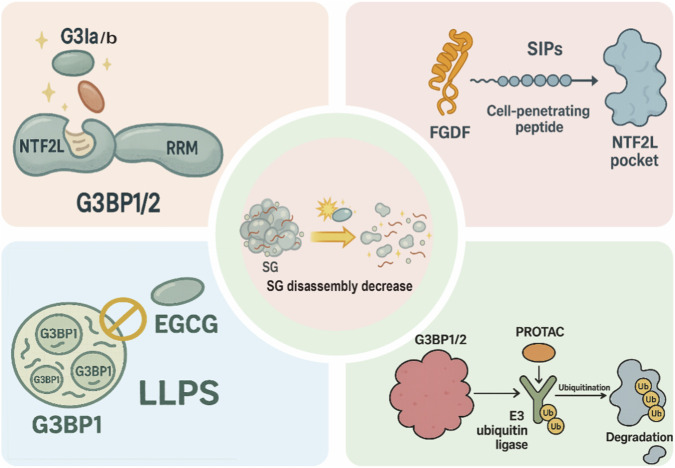
Pharmacological strategies targeting G3BP1/2 to modulate stress-granule (SG) disassembly. Top left: NTF2L pocket–binding small molecules G3Ia/G3Ib engage the NTF2L domain of G3BP1/2 and alter NTF2L–RRM conformational coupling. Top right: stress-granule–interfering peptides (SIPs), consisting of an FGDF motif fused to a cell-penetrating peptide, bind the NTF2L pocket and compete with endogenous interaction partners. Bottom left: the natural product EGCG perturbs G3BP1 liquid–liquid phase separation (LLPS), thereby changing the formation and material properties of G3BP1 condensates. Bottom right: G3BP1/2-directed PROTACs recruit E3 ubiquitin ligases to G3BP1/2, leading to their ubiquitination and proteasomal degradation. Central schematic: together, these structurally distinct modalities reshape the assembly–disassembly equilibrium of G3BP1-centred SGs, here illustrated as a net decrease in SG disassembly.

In addition, several recent studies on inflammation and tumor adaptive stress have employed G3Ia as a “chemical genetic tool” to selectively block G3BP-dependent SGs, thereby distinguishing SG-mediated cytoprotective effects from cell death signaling. These findings provide an important basis for further optimization of G3Ia and related compounds as lead molecules for drug development (Jia et al., 2024; Zhou et al., 2025). Overall, G3Ia and G3Ib represent the first structurally validated small-molecule inhibitors that directly target the NTF2L pocket of G3BP1/2. Although they are currently used primarily as mechanistic research tools, they provide a clear structural template for subsequent optimization in terms of pharmacokinetics and toxicity (Jia et al., 2024; Li J. et al., 2024; Parker et al., 2025; Buchan, 2024; Wang et al., 2025; Xing et al., 2023) (Figure 3; Table 1).

**TABLE 1:** Reported G3BP inhibition strategies and comparison with control tools.

Category	Representative molecules	Target/Binding site	Cellular effects	*In vivo* evidence	Advantages	Limitations	Recommended use
Small-molecule NTF2L pocket antagonists	FAZ-3532 (G3Ia), FAZ-3780 (G3Ib)	Bind FGDF pocket in G3BP1/2 NTF2L domain; compete with Caprin-1/USP10/nsP3 (Freibaum et al., 2024; Yang et al., 2024; Schulte et al., 2023)	Pre-stress: block SG nucleation; post-stress: dissolve SGs; multi-stressor, multi-line activity; minor impact on bulk translation (Freibaum et al., 2024)	Only cell/biochemical data; no animal efficacy yet (Freibaum et al., 2024)	Site-specific; prevent + dissolve SGs; mechanism clear (Freibaum et al., 2024)	Bulky and polar; CNS exposure unknown; G3BP1/2 selectivity needs improvement	First-choice probes for mechanism studies; leads for ADME/CNS and selectivity optimization
Peptides/peptidomimetics (FGDF/Caprin-1/USP10-derived)	TAT-SIP-C1/C2, SIP-U1-Antp	Caprin-1/USP10-FGDF mimetics that bind G3BP NTF2L and block PPIs (Li J. et al., 2024)	Reduce arsenite/drug-induced SGs; increase sorafenib sensitivity in HeLa (Li J. et al., 2024)	No systemic *in vivo* efficacy data yet (Li J. et al., 2024)	High affinity; precise binding-site mapping (Li J. et al., 2024)	Poor stability and delivery; possible immunogenicity; costly synthesis	*In vitro* site validation and combo-sensitization; templates for small molecules or PROTACs
Targeted degradation (PROTAC)	PT-129 (G3BP1/2 PROTAC)	NTF2L-based PROTAC that recruits E3 ligase to degrade G3BP1/2 (Dong et al., 2024)	Depletes G3BP1/2; prevents and clears SGs; blocks CAF-driven tumor growth (Dong et al., 2024)	In mice, inhibits tumor growth and the ATF4–migracytosis axis (Dong et al., 2024)	Destroys protein rather than just occupying site; useful for hard PPIs (Dong et al., 2024)	Very large; limited permeability/tissue distribution; CNS targeting difficult; off-target degradation risk	Proof-of-concept and mechanism work; test synergy with radio/chemotherapy or antimetabolites
Natural small-molecule inhibitors	EGCG	High-affinity G3BP1 ligand at Ras-GAP–binding and glycine-rich regions; disrupts G3BP1–RasGAP and G3BP1–cGAS.	Inhibits growth and soft-agar colonies in G3BP1-high H1299/CL13; weaker in G3BP1-low H460; suppresses Ras–MEK–ERK (Shim et al., 2010)	In AGS models and patient cells: blocks G3BP1–cGAS, reduces type I IFN and autoinflammation (Liu et al., 2019)	Direct G3BP1 target; rich PK/tox and oral safety data; tumor and autoinflammatory potential	Multi-target polyphenol; low oral exposure; rapidly metabolized	Chemical probe for G3BP1–RasGAP/cGAS; sensitizer in G3BP1-high tumors; scaffold for improved EGCG analogues

#### 4.1.2 PROTAC degraders: PT-129 and G3BP1/2-targeting PROTACs

Building on the systematic screening of a G3BP1/2-targeting ligand-1, Dong et al. (2024) linked this warhead to a thalidomide-derived cereblon ligand *via* a flexible PEG linker to generate the first G3BP1/2-targeting PROTAC molecule, PT-129, which bridges G3BP1/2 to an E3 ubiquitin ligase to induce their selective ubiquitination and degradation. In multiple cancer cell lines, PT-129 reduces G3BP1 and G3BP2 protein levels in a dose-dependent manner; according to vendor and patent data, its DC_50_ values are approximately 5 μM for G3BP1 and 10 μM for G3BP2, with minimal effects on non-target proteins, consistent with the characteristic profile of PROTAC-mediated target-selective degradation. Functionally, rapid depletion of G3BP1/2 by PT-129 suppresses SG formation and promotes the dissolution of pre-existing SGs, thereby blocking the ATF4-dependent migracytosis signaling axis and markedly attenuating cancer-associated fibroblast (CAF)–driven tumor cell migration and invasion. In mouse subcutaneous and orthotopic tumor models, systemic administration of PT-129 lowers G3BP1/2 levels and SG marker signals in tumor tissues, accompanied by reduced tumor growth and metastatic burden, suggesting that “reprogramming stress-adaptation networks *via* degradation of SG hub proteins” may represent a potential anticancer strategy (Dong et al., 2024; Lior et al., 2024). At present, PT-129 remains at an early experimental stage, and its *in vivo* pharmacokinetics, safety profile, and optimal dosing regimens have yet to be systematically evaluated. Nevertheless, this study provides initial proof of concept for using G3BP1/2 as PROTAC degradation targets and outlines a paradigm for strategies aimed at reducing SG hub proteins (Liang et al., 2020) (Figures 1B, 3; Table 1).

#### 4.1.3 Peptidic inhibitors: Stress granule inhibitory peptides (SIPs) and virus protein–derived peptides

Li J. et al. (2024) excised short peptide fragments containing FGDF motifs from the SG core proteins Caprin1 and USP10, fused them to cell-penetrating peptides such as TAT or Antennapedia, and generated two classes of SIPs, including TAT-SIP-C1/2 and SIP-U1-Antp. These SIPs bind the NTF2L domain of G3BP1/2 with high affinity and competitively block the interactions between Caprin1/USP10 and G3BP. In cells treated with arsenite or various SG-inducing anticancer agents (including sorafenib), SIP treatment markedly reduces cytoplasmic condensation of core SG proteins such as G3BP1/2, Caprin1, and TIAR, thereby suppressing SG assembly, while having only minor effects on basal translation rates and cell viability (Li J. et al., 2024; Adjibade et al., 2015). In hepatocellular carcinoma and other models, SIPs relieve SG-mediated cytoprotection and markedly increase sorafenib-induced cell death, suggesting that combining “SG inhibition + targeted therapy” may help overcome certain forms of chemoresistance (Li J. et al., 2024). Viruses themselves also extensively exploit FGDF/FGxF motifs to bind G3BP1/2 with high affinity and hijack SG components. For example, alphaviral nsP3, HSV-1 ICP8, and the SARS-CoV-2 N protein all engage the NTF2L domain binding site to competitively suppress SG assembly, thereby promoting viral replication and immune evasion (Panas et al., 2015; Yang et al., 2024). (Table 1; Figure 3).

Li’s study provides a broader perspective on targeting G3BP1, but additional limitations include:

Lack of SG disassembly analyses: This study mainly focuses on blocking SG assembly and does not systematically examine SG dissolution/disassembly dynamics after SGs are pre-formed.

No *in vivo* validation: The conclusions are based largely on *in vitro*/cell-based assays, and *in vivo* efficacy, pharmacokinetics, and safety of SIPs remain to be established.

### 4.2 Natural-product inhibitors: EGCG

Epigallocatechin-3-gallate (EGCG), the most abundant catechin in green tea, is a well-recognized antioxidant that reduces free radical–induced lipid peroxidation (Halder et al., 2021). In a CCl4-induced rat hepatotoxicity model, EGCG—alone and more potently in combination with silymarin—reduced serum ALT, alleviated oxidative stress (↓MDA, ↑GSH), suppressed pro-inflammatory signaling (TNF-α/NF-κB/IL-1β/TGF-β), and downregulated pro-fibrogenic pathways (p-ERK and p-SMAD1/2), supporting hepatoprotective and anti-fibrotic effects (Mostafa-Hedeab et al., 2022). Beyond these systemic actions, EGCG has also been reported to directly bind G3BP1 and disrupt its interaction with Ras GTPase-activating protein 1 (RasGAP), thereby dampening Ras/MAPK signaling and inhibiting soft-agar colony formation in lung cancer cells with high G3BP1 expression (Shim et al., 2010; Saeki et al., 2018; Cheng et al., 2020). Subsequent basic and translational studies, as well as several reviews, have consistently shown that binding of EGCG to G3BP1 attenuates downstream MEK/ERK activation and inhibits the proliferation of multiple solid tumor cell types, providing independent validation for EGCG as a multi-target natural product with preferential activity toward G3BP1 (Cheng et al., 2020; Zhang L. et al., 2022). In the context of innate immunity, Liu et al. (2019) found that G3BP1 forms a complex with cGAS and promotes its binding and phase separation with dsDNA, thereby enhancing cGAS–STING–mediated type I interferon production. As a G3BP1 inhibitor, EGCG disrupts pre-formed G3BP1–cGAS complexes, suppresses DNA-triggered cGAS activation and IFN production, and markedly reduces interferon-stimulated gene expression in cells and mouse models of Aicardi–Goutières syndrome (Liu et al., 2019). Subsequent studies by Zhao et al. (2022) and others further demonstrated that EGCG, by inhibiting G3BP1-mediated pre-condensation of cGAS, attenuates excessive cGAS–STING activation across multiple DNA-stimulated contexts, and has therefore been classified in several cGAS–STING reviews as a representative natural product that suppresses cGAS activation *via* the G3BP1 axis (Li et al., 2021; Huang et al., 2023). In the fields of ageing and inflammation, Omer et al. (2020) reported that genetic loss or pharmacological inhibition of G3BP1 (including EGCG treatment) diminishes cGAS-driven secretion of inflammatory senescence-associated secretory phenotype (SASP) factors without affecting the senescence program itself, suggesting that the “G3BP1–cGAS axis” may represent an attractive target for controlling inflammaging and the tumor microenvironment (Zhao et al., 2022; Omer et al., 2020; Zhu et al., 2022). Collectively, current evidence indicates that EGCG exerts its inhibitory effects through at least two G3BP1-related pathways: (i) disrupting the G3BP1–RasGAP interaction to attenuate oncogenic Ras/MAPK signaling, and (ii) suppressing G3BP1-dependent cGAS activation to alleviate type I interferon–driven autoinflammation, thereby suggesting potential relevance in both cancer and autoinflammatory diseases (Liu et al., 2019; Shim et al., 2010; Omer et al., 2020; Zhang M. et al., 2022) (Figure 3).

## 5 Direct agonist modulators of G3BP1/2

Compared with negative modulators that inhibit or degrade G3BP1/2, reports of agents that directly enhance G3BP1/2 function and promote SG assembly are much fewer. At present, the most robust evidence falls into two categories: (i) chiral small molecules that bind directly to the NTF2L pocket and induce the formation of G3BP1-positive condensates in the absence of exogenous stress or under mild stress; and (ii) natural products exemplified by perillaldehyde, icariin, and procyanidin B3, which have been reported to confer neuroprotective or anti-ischemic effects in models of neurodegeneration or brain ischemia *via* G3BP1-driven SG assembly (Park et al., 2024).

### 5.1 NTF2L pocket small-molecule agonists: enantiomeric compounds

**FIGURE 4:**
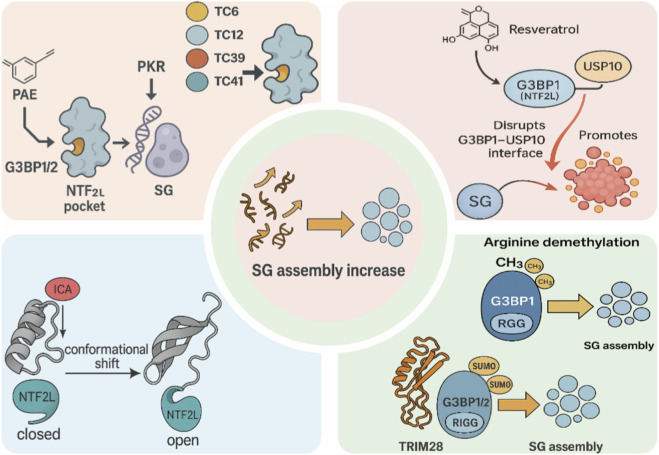
Endogenous and pharmacological mechanisms that enhance G3BP1-dependent stress granule (SG) assembly. Top left: Natural small molecules PAE and NTF2L-pocket agonists TC6/12/39/41 bind the NTF2L domain of G3BP1/2 and, in part *via* PKR activation, promote G3BP1-driven SG nucleation. Top right: Resveratrol binds the NTF2L region of G3BP1 and perturbs the G3BP1–USP10 interface, thereby shifting USP10 away from G3BP1 and facilitating SG formation. Bottom left: ICA induces a conformational transition of the G3BP1 NTF2L domain from a “closed” to an “open” state, favoring G3BP1 oligomerization and condensation. Bottom right: Post-translational modifications of the C-terminal RGG region of G3BP1/2, including arginine demethylation and TRIM28-mediated SUMOylation, further enhance G3BP1/2 self-association and LLPS, collectively driving increased SG assembly (center).

Building on the NTF2L pocket framework described in Section 2.3.1, recent studies have demonstrated that this same groove can be exploited not only for antagonism but also for small-molecule agonism, enhancing G3BP1/2 phase-separation capacity and SG assembly under specific cellular contexts. Cho et al. (2024) used the G3BP1 NTF2L structure for docking-based screening, identified several small molecules capable of binding NTF2L, and demonstrated that a subset of these candidates (TC6,TC12,TC39) can induce cytoplasmic G3BP1-positive granules even in the absence of exogenous stress. This process is largely independent of eukaryotic initiation factor 2α (eIF2α) Ser51 phosphorylation, indicating that “small-molecule–driven G3BP1 condensation” constitutes a regulatory route distinct from the canonical ISR. Subsequently, Park et al. (2024) constructed a chiral compound library and combined virtual docking with functional screening to obtain a series of enantiomers exemplified by TC41. TC41 engages the G3BP1 NTF2L pocket and induces SG-like granules containing RFP–G3BP1 and endogenous G3BP1 in HEK293, NIH3T3 and U2OS cells, accompanied by increased eIF2α phosphorylation, suggesting that it promotes SG assembly through a cooperative mechanism that couples direct targeting of G3BP1 with partial activation of the ISR. Based on docking poses and functional differences among individual enantiomers, the authors further proposed that distinct stereoisomers built on the same chiral scaffold could, in principle, bias toward either stabilizing or disrupting the G3BP1 dimer, thereby enabling stereoselective modulation of SG-promoting *versus* SG-inhibiting effects at the same NTF2L pocket and providing a conceptual framework for developing both G3BP1 agonists and inhibitors from a shared chemical backbone (Park et al., 2024). Together with broader drug-design efforts targeting biomolecular condensates and intrinsically disordered proteins (IDPs), these findings suggest that the NTF2L pocket is not limited to inhibitory ligands. With stereochemical tuning and optimization of key interactions, small molecules may be engineered to enhance G3BP1 phase separation and promote SG phenotypes. Overall, this work supports NTF2L-pocket–centric strategies for modulating the G3BP1–SG axis. (Jia et al., 2024; Qin et al., 2025) (Table 2; Figure 4).

**TABLE 2:** Reported G3BP1/2 agonists/positive modulation strategies.

Category	Representative molecules	Target/Site	Cellular effects	*In vivo* evidence	Advantages	Limitations	Recommended use
NTF2L domain small-molecule agonists	TC6, TC12, TC39, TC41 (Park et al., 2024; Cho et al., 2024)	G3BP1 NTF2L FGDF pocket (Cho et al., 2024)	Induce G3BP1^+^ SG-like granules without added stress; some ↑p-eIF2α or p-S6K (Park et al., 2024)	Only cell-based data; no animal efficacy yet (Park et al., 2024)	First NTF2L positive modulators; show bidirectional SG control from same pocket (Park et al., 2024)	Long-term safety and effects in disease models unknown (Park et al., 2024)	Chemical probes for G3BP1 conformation–SG assembly; leads for neuroprotective drug design (Park et al., 2024)
Natural-product agonists	Perillaldehyde (PAE) (Fang et al., 2025)	Binds G3BP1/2; promotes G3BP-dependent SG assembly; modulates HDAC6 (Fang et al., 2025)	↑G3BP1^+^ SG and p-eIF2α; ↓α-syn aggregates and apoptosis in DA neurons (Fang et al., 2025)	Improves motor behavior and DA neuron survival in worm and PD mouse models (Fang et al., 2025)	Most complete G3BP-dependent SG agonist; neuroprotective and anti-aggregation together (Fang et al., 2025)	Multi-target natural product; human PK and long-term safety unclear (Fang et al., 2025)	Lead for PD and protein-aggregation diseases; test ‘SG enhancement is beneficial’ concept (Fang et al., 2025)
	Icariin (ICA) (Li et al., 2025)	Binds G3BP1 NTF2L domain; shifts closed→open; boosts SG assembly and IGF2BP1 binding (Li et al., 2025)	Under ischemia/oxidative stress: ↑G3BP1^+^ SG/condensates, activates m^6^A–IGF2BP1, stabilizes protective mRNAs, ↓ROS and cell death (Li et al., 2025)	In MCAO mice, reduces infarct size and improves deficits; G3bp1 or Igf2bp1 knockdown weakens protection (Li et al., 2025)	Shows that reshaping G3BP1 condensates promotes protective SGs; supports G3BP-targeted neuroprotection with PAE (Li et al., 2025)	Multi-target flavonoid; non-G3BP targets and long-term safety not fully defined; mainly stroke data so far (Li et al., 2025)	Lead for ‘enhance protective G3BP1 condensates’ in acute brain injury; tool to dissect G3BP1–m^6^A–IGF2BP1–SG topology (Li et al., 2025)
	Procyanidin B3 (PB3) and derivatives (e.g., PB3-6c) (Zhang et al., 2025)	G3BP1 NTF2L domain near R107 (Zhang et al., 2025)	Stabilize SGs, inhibit SG disassembly, enhance G3BP1 LLPS; lessen oxidative-stress-induced apoptosis (Zhang et al., 2025)	In ischemic stroke mice: reduce neuronal death and improve motor function (Zhang et al., 2025)	Polyphenol with good safety; BBB-penetrant; antioxidant/anti-inflammatory synergy (Zhang et al., 2025)	Low oral bioavailability; rapid metabolism; SAR not fully mapped (Zhang et al., 2025)	Neuroprotection, anti-ischemic brain injury, potential neurodegeneration therapy (Zhang et al., 2025)
​	Resveratrol (Amen et al., 2021)	Directly binds G3BP1 (NTF2L region); perturbs the G3BP1–USP10 interface and favors SG assembly under stress (Amen et al., 2021)	Induces G3BP-dependent stress granule assembly with unusually rapid clearance; alleviates G3BP1-mediated repression of USP10–p53, stabilizing p53 and triggering p53-dependent apoptosis; can remodel SG composition and stress-response signaling (Amen et al., 2021)	Exhibits anti-tumor and cytoprotective activities in multiple preclinical models and has early clinical PK/safety data; SG/G3BP1-focused pharmacodynamic readouts remain limited (Amen et al., 2021)	Proof-of-concept natural agonist of the G3BP–SG axis; mechanistically defines the G3BP1–USP10–p53 linkage; rich stilbene SAR/PK literature and tractable scaffold for tuning SG dynamics (Amen et al., 2021)	Strongly pleiotropic with numerous off-target pathways; low oral bioavailability and marked first-pass metabolism; *in vivo* G3BP1/2 target engagement and SG modulation not yet rigorously quantified	Chemical probe for G3BP1-centered SG activation and the G3BP1–USP10–p53 axis; starting scaffold for designing more selective G3BP1/2-directed SG agonists or PROTAC warheads with improved PK and specificity

In U2OS cells (2 h, 5 µM), SG induction ranked TC6/TC39 > TC41 > TC12. When normalized to the study-specific thapsigargin (Tg, 1 µM) positive control, TC6/TC39/TC41 showed similar relative activity (∼35–36% of Tg), whereas TC12 remained clearly weaker (∼21% of Tg) (see Table 3 for full numerical values).

**TABLE 3:** Relative SG-inducing activity of TC compounds normalized to the thapsigargin (Tg) positive control in U2OS cells (2 h).

Compound	Study	SG% (drug)	SG% (Tg)	Relative-to-Tg (%)
TC6	Cho et al. (2024)	31.9	88.4	36.1
TC39		31.2	88.4	35.3
TC12		18.8	88.4	21.3
TC41	Park et al. (2024)	25.5	71.7	35.6

SG% (drug) indicates the percentage of SG-positive cells measured after treatment with each compound (5 µM) for 2 h in U2OS, cells. Tg (1 µM) was used as the positive control within each study. Relative-to-Tg (%) was calculated as (SG%_drug/SG%_Tg) × 100. Values were extracted from the reported data in Cho et al. (2024) and Park et al. (2024).

The differences in the apparent potency among these four compounds can be explained on two levels of evidence:

Differences in binding strength and the contact network within the NTF2L pocket (which sets the intrinsic efficacy ceiling). Docking analyses suggest stronger binding energies for TC6 and TC39, with TC6 (−9.5 kcal/mol) > TC39 (−9.0 kcal/mol) >> TC12 (−6.8 kcal/mol). In addition, TC39 engages a more extensive set of contacting residues (seven residues), whereas TC12 contacts only four residues. This implies that TC39 may better stabilize key hydrophobic and π-interaction networks within the NTF2L pocket, thereby more effectively promoting G3BP1 granule assembly and/or maintenance (Table 3).

Differences in the induction pathways—eIF2α–ISR *versus* mTOR–S6K *versus* cytotoxic stress (which shapes kinetics and granule size). For TC6/TC12/TC39 (Cho et al. 2024), these compounds show little to no induction of p-eIF2α at 2 h, suggesting that they do not primarily act through the canonical ISR pathway. In contrast, TC12 and TC39 increase p-S6K, leading the authors to propose that they may promote assembly *via* the mTOR–S6K axis. A further key distinction is that TC6 inhibits U2OS cell growth in a dose-dependent manner (consistent with cytotoxicity or additional stress), whereas TC12/TC39 show no detectable cytotoxicity at ≤10 µM. On this basis, the authors speculated that TC6 may introduce a distinct type of cellular stress, thereby driving stronger granule formation and/or amplification. This also explains why TC6 and TC39 yield similar percentages of SG-positive cells, yet TC6 produces a markedly higher granule area readout (granule index) (TC6 = 5.5 vs TC39 = 1.3; TC12 = 1.1): TC6 may preferentially promote granule enlargement/expansion rather than merely increasing the number of cells exhibiting small granules.

For TC41 (Park et al. 2024), the mechanism appears closer to a canonical ISR, eIF2α-dependent mode of action and is influenced by stereochemistry. Park et al. (2024) explicitly reports that TC41 induces G3BP1-positive granules through eIF2α phosphorylation rather than the S6K pathway, and that integrated stress response inhibitor (ISRIB) effectively suppresses TC41-induced granules, supporting a stronger dependence on ISR signaling. The study further shows that nine stereoisomers of TC41 differ in docking energies and hydrogen-bond/hydrophobic interaction patterns with the NTF2L pocket (affinity range approximately −8.4 to −9.4 kcal/mol, with the strongest stereoisomer reaching −9.4 kcal/mol). Variations in how different stereoisomers mimic key “FGDF-like” interactions (e.g., involving N122/F124) may lead to differences in the fraction of active conformers or in conformational matching, resulting in a moderate induction by TC41 (25.5%) under the same dose and short time window.

### 5.2 Perillaldehyde (PAE): A G3BP1-dependent natural SG agonist

Fang et al. (2025) reported in Parkinson’s disease (PD)–related models that the natural monoterpene PAE markedly ameliorates PD-like motor deficits in α-syn–overexpressing *C. elegans* and MPTP-treated mice, and reduces α-syn aggregation in dopaminergic neurons of the substantia nigra. Mechanistically, PAE directly binds to G3BP1, as supported by *in vitro* SPR measurements, and robustly enhances G3BP1/gtbp-1–dependent SG assembly in neurons and *C. elegans*, promotes eIF2α phosphorylation, then improves protein quality control by modulating HDAC6 and microtubule-associated deacetylation processes, thereby alleviating mitochondrial damage and neuronal apoptosis. In *C. elegans*, knockdown of gtbp-1 or tiar-1, or pharmacological inhibition of SG assembly, markedly blunts the locomotor improvement and neuroprotective effects of PAE, supporting the notion that its key efficacy depends on the G3BP family–mediated SG pathway (Fang et al., 2025; Fang et al., 2024). Conversely, in cGAS–STING–related autoinflammatory models, Chu et al. (2021) reported that perillaldehyde (PAH) can directly inhibit the DNA binding and enzymatic activity of cGAS, reduce dsDNA-induced IFN-β and ISG expression, and markedly attenuate type I interferon–driven immune activation in TREX1-deficient and other Aicardi–Goutières syndrome (AGS)-related mouse models (Chu et al., 2021; Wei et al., 2024) (Table 2; Figure 4).

### 5.3 Icariin (ICA) and procyanidin B3 (PB3): Natural agonists that remodel the topology of G3BP1 condensates

Using a G3BP1-centred multi-omics and structural biology strategy, Li et al. (2025) recently identified the flavonoid ICA from a natural-product library as a small-molecule inducer that directly promotes G3BP1 condensate formation. Their study showed that ICA interacts selectively with the N-terminal NTF2L domain of G3BP1, shifting it from a “closed” to a more oligomerization-competent “open” conformation, thereby markedly enhancing the phase-separation capacity of G3BP1 and its SG-nucleating potential. Topological analyses further revealed that ICA-induced G3BP1 SG assembly selectively recruit the m^6^A reader IGF2BP1 to form a G3BP1–IGF2BP1–m^6^A “epitranscriptomic hub”, which, by activating an m^6^A-dependent AMPK–MAPK–GPX4 axis, increases cellular tolerance to oxidative stress and ferroptosis. In ischemia-related models, ICA significantly reduces infarct volume, improves neurological deficit scores, and attenuates blood–brain barrier disruption and neuronal apoptosis in both *in vitro* oxygen–glucose deprivation/reperfusion (OGD/R) assays and *in vivo* middle cerebral artery occlusion models. Knockdown of G3BP1 or perturbation of IGF2BP1 markedly diminishes the protective effects of ICA, demonstrating that its neuroprotection depends on G3BP1-mediated SG remodelling and the m^6^A–IGF2BP1 axis (Li et al., 2025) (Table 2).

In parallel, Zhang et al. (2025) identified G3BP1 as a key target of PB3 and its derivatives. PB3 restrains excessive SG degradation and reduces neuronal apoptosis, thereby significantly mitigating neural damage in both *in vitro* and *in vivo* models of cerebral ischemia. On the basis of the PB3 scaffold, the authors synthesized and screened a series of derivatives, among which compound 6c exhibited both stronger neuroprotective activity and improved blood–brain barrier permeability, providing a structural starting point for G3BP1-targeted lead compounds for ischemic stroke (Zhang et al., 2025).

Taken together, ICA and PB3 exert clearly G3BP1-dependent neuroprotective effects in ischemic stroke models—by enhancing G3BP1-mediated SG assembly and by inhibiting SG degradation, respectively. Along with PAE in PD models, they currently represent the three best-supported classes of “natural SG agonists that directly target G3BP1” (Figure 4).

PB3 induces only a modest SG response in the HT22 OGD/R (24 h) model, consistent with limited amplitude and relatively low apparent potency under this severe stress paradigm and time window (Table 4).

**TABLE 4:** Comparison of SG-inducing activity of PB3 and ICA under their respective experimental conditions.

Compound	Study	Experimental condition (cell/model/time)	Doses (µM)	Baseline SG+ (%) at 0 µM	Max SG+ (%)	ΔEmax (pp)	EC20 (µM)	EC50 (µM)
PB3	Zhang et al. (2025)	HT22/OGD/R/24 h	0, 20, 50, 100	1.91	9.88	7.97	11.98	35.00
ICA	Li et al. (2025)	HEK293T/none/0 h	0, 5, 10, 20	8.30	9.40	1.10	1.00	2.50
		HEK293T/none/6 h	0, 5, 10, 20	8.00	23.10	15.10	4.19	7.74
		HEK293T/none/12 h	0, 5, 10, 20	13.40	32.90	19.50	3.61	8.57
		HEK293T/none/24 h	0, 5, 10, 20	14.80	53.10	38.30	6.64	12.63

*EC20 and EC50 were defined as the concentrations required to reach 20% and 50% of the maximum incremental effect (ΔEmax) within the same experimental condition. First, the incremental effect at each dose was calculated as ΔE(dose) = SG+(dose) − SG+(0 µM). Then, ΔEmax, was obtained as the maximal ΔE, across the tested doses. EC20 and EC50 were estimated by linear interpolation between the two neighboring doses that bracket the target ΔE, level (0.2 × ΔEmax, or 0.5 × ΔEmax). Units are µM. Baseline SG+ (%): percentage of SG-positive cells at 0 µM (vehicle). Max SG+ (%): highest observed SG-positive percentage among tested doses for the same condition. Emax (pp): Max SG+ (%) − Baseline SG+ (%), expressed in percentage points (pp).

In contrast, ICA shows a clear time-dependent enhancement of SG induction in HEK293T cells, with efficacy increasing progressively over time and apparent potency in the low–mid µM range (Table 4).

Together, these data support the qualitative conclusion that PB3 is modest, whereas ICA strengthens over time and appears more active under these conditions.

The likely reasons are as follows:

PB3 may act more as a “stabilizer/inhibitor of SG clearance,” such that its apparent induction is constrained in a strong-stress model. In the HT22 OGD/R system, the authors explicitly distinguished “promoting assembly” from “inhibiting degradation” and provided key evidence: short-term PB3 treatment (1 h) did not significantly change the SG-positive fraction, whereas SG positivity increased at 24 h. Based on this, they proposed that PB3 primarily inhibits SG degradation rather than rapidly triggering SG assembly. In addition, PB3 modulated eIF2α-pathway factors related to SG dynamics (changes in kinases/phosphatases involved in eIF2α phosphorylation), thereby stabilizing SGs during the degradation phase. PB3 also enhanced G3BP1-associated mRNA levels, supporting a mechanism in which PB3 stabilizes SG cores/components and reduces disassembly.

ICA is a direct inducer of G3BP1 phase separation/nucleation, which more readily yields a higher ΔEmax and time-dependent accumulation. The ICA study clearly identified ICA as a G3BP1 phase-separation inducer, showing time- and dose-dependent increases in intracellular condensates/granules in HEK293T cells. Importantly, ICA does not rely on upstream eIF2α phosphorylation signaling (p-eIF2α, PKR, PERK, *etc.*, were unaffected), and the authors suggested that it more directly participates in triggering of G3BP1-positive SGs. Mechanistically, ICA promotes G3BP1 dimerization/oligomerization *via* the NTF2L domain, thereby driving LLPS, and a “close-to-open” allosteric model was proposed in which ICA relieves IDR1-mediated inhibition of NTF2L to promote higher-order oligomerization and droplet formation.

### 5.4 Resveratrol (RSV)

Resveratrol is a plant-derived stilbene compound. Previous studies have reported that resveratrol engages the NTF2L domain of G3BP1 *via* hydrophobic interactions with Val11, Phe33, and Phe124, and that its hydroxyl group forms a hydrogen bond with the side chain of Gln18 (Oi et al., 2015). Amen et al. (2021) performed a screen for small molecules that induce stress granule (SG) formation and identified the resveratrol analogue piceatannol as an SG inducer. They therefore investigated the SG-inducing activities of resveratrol and a panel of natural and synthetic analogues. The results showed that resveratrol, piceatannol, pterostilbene, and 3,4,5,4′-tetramethoxystilbene can each induce SG formation at concentrations of 100–400 μM. Amen et al. (2021) demonstrated that resveratrol (and close analogues) trigger the formation of SGs in mammalian cells only when G3BP1/2 are present, indicating these proteins are required for resveratrol’s SG-inducing effect. Unlike classical stressors (e.g. arsenite) that induce persistent SGs, resveratrol-induced granules exhibit atypically rapid clearance, suggesting qualitative differences in their assembly or stability (Amen et al., 2021). Notably, resveratrol-treated cells form SGs despite inducing only modest phosphorylation of eIF2α (the canonical translational shutdown pathway for SG assembly) (Saha et al., 2025). This implies that resveratrol engages non-canonical mechanisms of SG assembly, relying less on global translation arrest and more on direct modulation of SG nucleator proteins.

Mechanistic studies reveal that resveratrol directly binds to G3BP1, affecting its ability to interact with binding partners. In particular, resveratrol binding prevents the association of G3BP1 with USP10, an FGDF-motif protein that normally binds G3BP1’s NTF2L domain and can suppress spontaneous SG aggregation (Kang et al., 2021). By blocking the G3BP1–USP10 interaction, resveratrol frees G3BP to engage in multimeric self-association and RNA binding, activities crucial for nucleating SGs. Biochemically, resveratrol binding appears to “reduce the protein–protein association valency” of G3BP1– i.e. limiting certain G3BP1 interactions while permitting others. This change in valency is proposed to drive the formation of SGs that are more transient: G3BP1 still nucleates SG assembly, but the resulting granules disassemble faster, likely due to fewer stable cross-links in the SG matrix (Amen et al., 2021). Consistently, enhancing G3BP’s propensity to phase-separate (for example, by disrupting its interaction with USP10 or by dephosphorylating Ser-149) is known to promote SG assembly (Kang et al., 2021). Thus, resveratrol’s physical interaction with G3BP1/2 serves as a molecular trigger, promoting G3BP-mediated condensation of SG components (Fan et al., 2025) (Figure 4).

This paper has several limitations. First, the effective concentrations are relatively high (100–500 μM), and the authors explicitly note in the Methods that conditions need to be optimized for different cell types; moreover, resveratrol has limited stability in solution.

In addition, the evidence for “direct binding” in this study is largely inherited from prior literature and supported indirectly by functional readouts. Much of the proposed mechanism relies on earlier work suggesting that resveratrol can engage a G3BP-related interface and displace USP10, rather than providing new quantitative binding measurements in the current study (e.g., SPR, ITC, or NMR).

Taken together, these considerations prompt a focused discussion of specificity, therapeutic window, and delivery challenges.

## 6 Perspectives and challenges

This section highlights the main barriers to translation and the practical steps needed to turn G3BP–SG modulators into developable therapies.

### 6.1 Target specificity and off-target risk

**FIGURE 5:**
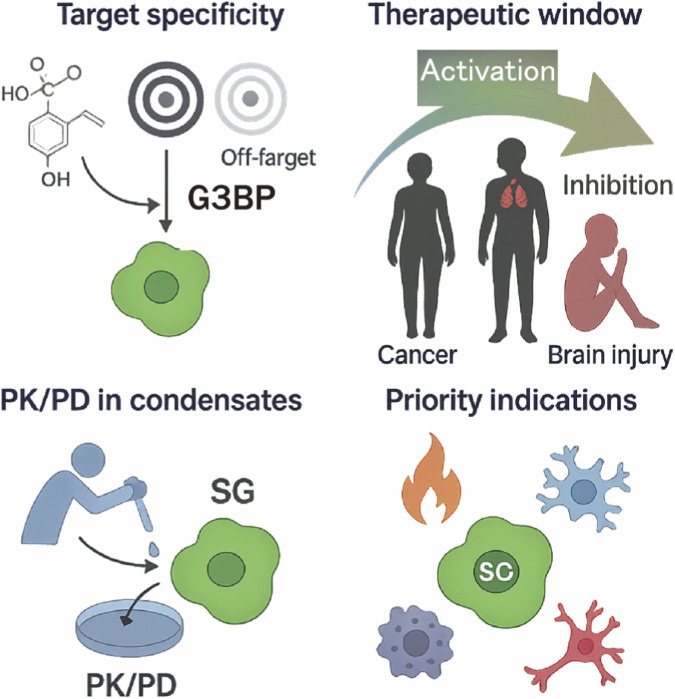
Perspectives and challenges for clinical translation of G3BP–SG axis modulators. Top left, target specificity and off-target risk: most currently available G3BP1/2-directed small molecules, particularly pleiotropic natural products, act on multiple antioxidant, metabolic and epigenetic pathways in addition to G3BP1/2, necessitating combined genetic (e.g., *G3bp1/2* knockout or NTF2L-mutant models) and pharmacological (e.g., G3BP1/2-targeting PROTACs) strategies to rigorously distinguish on-target from off-target effects. Top right, therapeutic window and tissue selectivity: inhibition of the G3BP–SG axis is expected to weaken stress adaptation in SG-addicted tumors, whereas activation of G3BP1 condensates may be beneficial in acute neurological injury, underscoring the need to tailor tissue distribution, CNS penetration and dosing schedules to disease context. Bottom left, PK/PD in condensates: SGs are prototypical biomolecular condensates in which drug partitioning, diffusion and binding kinetics differ markedly from bulk cytosol, calling for dedicated “condensate-phase PK/PD” frameworks that quantify intragranular exposure and target occupancy. Bottom right, priority indications for clinical translation: conceptually attractive entry points include autoinflammatory diseases linked to aberrant G3BP1–cGAS–STING signaling, therapy-resistant solid tumors with high G3BP1/2 and SG burden, and acute CNS disorders such as stroke or early Parkinson’s disease, where G3BP-centred precision interventions may be feasible.

Most currently available G3BP1/2-directed small molecules—particularly natural products such as EGCG, PAE, and ICA—are intrinsically multitarget agents. They modulate not only G3BP1/2 but also antioxidant, metabolic, and epigenetic pathways, complicating attribution of their pharmacological effects primarily to G3BP1/2 (Legeay et al., 2015; Alam et al., 2024; Li S. et al., 2024; Qiu et al., 2021; Chen et al., 2022; Xu et al., 2014; Wang et al., 2021; Cong et al., 2026). In future studies, it will be essential to integrate *G3bp1/2* knockout or NTF2L pocket–disrupting mutants with G3BP1/2-targeting PROTACs (e.g., PT-129) in animal models, and to systematically compare compound efficacy and toxicity under “on-target” *versus* “off-target” conditions. Such combined genetic and pharmacological approaches will be required to achieve rigorous target validation and pathway deconvolution (Hu Y. et al., 2025; Liu Z. et al., 2022) (Figure 5).

### 6.2 Inhibition *versus* activation: Therapeutic window and tissue selectivity

The seemingly opposite therapeutic logic of modulating the G3BP–SG axis in cancer *versus* the nervous system reflects fundamental differences in cellular goals, time scales, and subcellular organization. In tumors, SGs are often repeatedly and chronically engaged to buffer translational stress and to sustain pro-survival programs under hypoxia, nutrient deprivation, and therapy, creating an “SG-addicted” state in which sustained SG inhibition can selectively erode stress tolerance. In neurons and axons, by contrast, RNA granules and SG-like assemblies are integral to acute homeostatic buffering and compartmentalized RNA regulation (e.g., local translation and transport), such that transient enhancement of adaptive SG responses can be neuroprotective during acute insults, while persistent or dysregulated condensates can become maladaptive by promoting pathological aggregation and RNA dyshomeostasis. Therefore, the key variable is not simply “inhibit *versus* activate,” but when, where, and for how long G3BP1/2 condensates are modulated—highlighting the necessity of tissue-selective delivery and temporally controlled dosing.

#### 6.2.1 Cancer: Chronic stress adaptation and SG addiction → rationale for sustained inhibition

Inhibiting the G3BP–SG axis is expected to weaken tumor cells’ stress adaptation under hypoxia, nutrient deprivation, and chemotherapeutic pressure, and multiple recent cohort and mechanistic studies have linked high G3BP1 expression or aberrant G3BP1 processing to higher grade disease, poor prognosis, and therapy resistance in glioma, osteosarcoma, head and neck cancer, and other solid tumors (Li Q. J. et al., 2024; Chen et al., 2025; Dai et al., 2023; Shi et al., 2025). Together with cancer-focused studies demonstrating that G3BP1-driven SGs sustain leukemia stemness and chemoresistance in hematologic malignancies (Jia et al., 2024; Bi et al., 2025; Biancon et al., 2022; Jin et al., 2024). In these settings, G3BP1-centred SGs promote survival by buffering translation, stabilizing pro-survival and immune-evasive transcripts, and sustaining adaptive response programs under DNA damage or kinase inhibition (Jia et al., 2024; Chen et al., 2025).

Therapeutically, this vulnerability may be exploited through sustained or combination strategies: (i) direct inhibition or degradation of G3BP1/2 (e.g., small-molecule NTF2L pocket ligands or PROTAC degraders): to disrupt SG nucleation; (ii) co-targeting upstream stress-kinase pathways (such as eIF2α or MAPK signaling) to prevent SG assembly under therapy-induced stress; and (iii) combining SG inhibitors with chemotherapy, radiotherapy, or targeted kinase inhibitors to abrogate adaptive translational reprogramming and enhance tumor cell killing (Zhao et al., 2021; Dai et al., 2023; Bittencourt et al., 2019; Cho et al., 2019; Meng et al., 2025). Such approaches aim to convert chronic stress adaptation into an irreversible vulnerability, thereby overcoming resistance associated with SG-dependent survival programs.

#### 6.2.2 CNS/axon: Acute protection vs chronic maladaptation → rationale for temporally controlled activation or disassembly

In the nervous system, SGs and related RNA granules are best viewed as context- and time-dependent condensates rather than uniformly protective or pathogenic entities. Acute, transient, and reversible G3BP1/2-centered SG assembly can be adaptive by buffering translation, preserving RNA/protein homeostasis, and coordinating stress-response signaling during insults such as ischemia or toxin exposure. This adaptive framework is supported by pharmacological evidence: agents such as PAE and icariin (ICA) have been reported to enhance G3BP1-mediated SG assembly and to confer robust neuroprotection in toxin-based Parkinson’s disease models and cerebral ischemia models, respectively, in a largely G3BP1-dependent manner (Fang et al., 2025; Li et al., 2025).

However, under chronic stress or disease-protein burden, SG-like assemblies may become persistent and biophysically altered, leading to prolonged sequestration of essential factors, RNA dyshomeostasis, and facilitation of pathological protein aggregation (Yuan et al., 2025; Glineburg et al., 2024; Rhine et al., 2022). Importantly, neuronal polarity adds an additional layer: axon-localized G3BP1 granules can constrain local translation and regenerative programs, such that granule disassembly may promote axon growth and repair in specific injury contexts. For example, recent data show that disassembly of G3BP1-containing granules in adult CNS neurons can enhance axon growth and neural repair, suggesting that persistent G3BP1 condensates may act as a brake on regenerative capacity in some contexts (Sahoo et al., 2025). These observations imply that CNS-directed modulation of the G3BP–SG axis must be temporally and spatially controlled, favoring short, condition-specific “activation” for acute neuroprotection while avoiding sustained SG stabilization that could exacerbate chronic neurodegeneration or impede regeneration. Accordingly, the therapeutic direction (inhibit vs activate) should be defined by disease phase, cellular compartment, and intended endpoint (survival vs repair), rather than by target engagement alone. (Aramburu-Nunez et al., 2022; Pernin et al., 2024) (Figure 5).

### 6.3 Pharmacokinetics (PK)/pharmacodynamics (PD) issues in LLPS systems

SGs are prototypical biomolecular condensates, and the partition coefficients, diffusion behavior, and binding kinetics of small molecules within them differ substantially from those in homogeneous solution. Studies of nuclear and synthetic condensates have shown that drug concentrations within the condensed phase can differ from those in the surrounding cytosol by several orders of magnitude, with decisive impact on efficacy and toxicity. Yet, for small molecules targeting the G3BP–SG axis, a systematic quantitative framework for “condensate-phase PK/PD” is still lacking. Future work will need to combine single-molecule imaging, quantitative mass spectrometry, and multiscale simulations to determine the true intragranular exposure and target occupancy of candidate compounds within SGs, thereby providing a robust basis for dose selection and safety assessment (Figure 5).

### 6.4 Priority indications for clinical translation

Currently, the most clearly defined directions with feasible biomarker strategies include: (i) EGCG-derived compounds targeting the G3BP1–cGAS–STING axis in type I interferon–driven autoinflammatory diseases; (ii) G3Ia/G3Ib- or PT-129-based combinations with chemotherapy or targeted agents in “high G3BP1/2 + high SG burden” therapy-resistant solid tumors; and (iii) G3BP1 agonists such as PAE, ICA, and PB3 in acute cerebral ischemia and early PD, where acute stress and protein aggregation predominate. In these contexts, G3BP1/2 show clear pathological associations and allow patient stratification based on SG burden, cGAS–STING activity, or transcriptomic signatures, making them attractive candidates for future clinical studies of “G3BP–SG axis–centric precision interventions.” (Figure 5)

Based on these challenges, we propose practical recommendations for discovery pipelines, benchmarking, and reporting standards.

## 7 Recommendations for next-step drug development

### 7.1 Drug discovery: Promoting *versus* inhibiting condensation

As summarized in Section 2.3.1, engagement of the G3BP1/2 NTF2L pocket can support bidirectional modulation of stress granule (SG) phenotypes; however, a generalizable, quantitatively predictive framework that links ligand properties and cellular context to SG outcomes is still lacking. For example, if the goal is to develop an SG-condensation inhibitor, beyond engineering stable molecules that mimic the viral nsP3 FGDF motif to engage G3BP1/2, it remains unclear how to systematically identify and prioritize natural products with anti-condensation activity. Addressing this gap represents a major unmet need in the SG field and an urgent problem for translational drug discovery.

### 7.2 Drug discovery: Compound screening

Current efforts to discover and engineer G3BP1-targeting modulators can be organized into three complementary pipelines that differ by the entry point (phenotype-first *versus* structure-first) and by how target causality is established.

#### 7.2.1 From candidate G3BP1-targeting drugs to experimental validation

This route typically starts from a functional compound with a well-defined cellular and/or *in vivo* phenotype or therapeutic benefit. Chemical proteomics is then applied to converge the putative target(s) onto G3BP1, followed by multiple orthogonal validation layers to establish a closed “direct binding–binding site–functional causality” chain. PB3 and EGCG represent a canonical example of this strategy.

#### 7.2.2 Cell-based high-content screening (HCS) followed by target/structure confirmation

Here, imaging-based HCS is used to capture compounds that quantitatively perturb G3BP1/2 puncta or SG phenotypes (e.g., number, size, persistence, and dissolution kinetics). Hits are subsequently triaged by dose–response and stress-panel validation, and then subjected to target confirmation and binding-site mapping using biophysics and structural/domain-level validation (docking/crystallography where feasible, or mutational tests). ICA and PAE are representative examples of this phenotype-driven discovery path.

#### 7.2.3 Structure-based discovery centered on the NTF2L pocket

This route starts from the structural definition of druggable surfaces on G3BP1—most prominently the FGDF-binding groove within the NTF2L pocket—and applies pharmacophore modeling and/or docking-based virtual screening to prioritize top-ranked candidates. These are advanced through experimental binding validation (SPR/ITC) and, when needed, chemical-probe–enabled confirmation (e.g., photoaffinity labeling) before cell-based SG assays and lead optimization. TC6/TC41 illustrate docking-first discovery, while the #129→PAL-129→PT-129 progression exemplifies a more stringent structure-first workflow that couples large-scale *in silico* screening to chemical proteomics and, ultimately, PROTAC engineering. Mechanism-guided variants of this route include FGDF-inspired peptidomimetics (G3Ia/G3Ib) and interaction-competitor peptides (SIPs) derived from Caprin1/USP10/nsP3 interfaces.

Each of the three routes has distinct strengths and limitations. A phenotype-first target deconvolution strategy starts from a well-defined cellular or *in vivo* phenotype and therefore maximizes translational relevance. Cell phenotype–driven high-content screening (HCS) can systematically capture compounds that modulate stress granule (SG) assembly/disassembly across a broader chemical space, thereby expanding both SG biology and the landscape of actionable intervention nodes. In contrast, structure-first discovery centered on the NTF2L pocket uses a clearly druggable binding site as an anchor, facilitating well-resolved structure–activity relationships (SAR) and offering superior potential for selectivity engineering, developability, and iterative optimization.

From a drug-discovery standpoint—particularly in terms of operational feasibility and scalability—we suggest prioritizing two entry points: cell-based HCS and NTF2L pocket–centric structure-guided discovery. On one hand, these strategies generate large-scale, reusable screening datasets and hit collections that can be continuously mined and reanalyzed—methodologically analogous to the secondary exploitation of omics resources such as TCGA—thereby progressively improving hit quality and expanding accessible chemical space. On the other hand, starting from pre-existing “putative G3BP1-targeting” compounds and then performing target deconvolution and mechanistic substantiation, while potentially closer to translation, often carries greater mechanistic uncertainty (polypharmacology, indirect pathway effects, and undefined target engagement within LLPS microenvironments) as well as higher time and financial costs, ultimately reducing predictability and slowing iterative optimization.

### 7.3 Drug development: Selection of readouts

At present, studies assessing stress granules (SGs) after drug treatment use a wide variety of readouts and there is no unified standard. Consequently, direct comparisons across studies have become extremely difficult. For example, Freibaum et al. (2024) used total SG area as the primary metric, whereas Dong et al. (2024) used SG number per cell. In contrast, Li J. et al. (2024), Fang et al. (2025), Cho et al. (2024), Park et al. (2024), Amen et al. (2021), Li et al. (2025), and Zhang et al. (2025) quantified SGs as % SG-positive cells (Tables 5, 6). Overall, % SG-positive cells appears to be the most frequently used metric. We therefore recommend using % SG-positive cells as the primary endpoint, for the following reasons:

**TABLE 5:** Study details of G3BP1 inhibitors.

Study	Compound	Dose (μM)	%SG_reduction_pct	Index	Cell line
Freibaum et al. (2024)	G3Ia	5	36	Total SG area	U2OS, 20 min
		20	59		
		50	80		
	G3Ib	5	50		
		20	85		
		50	93		
Li Y. et al. (2024)	TAT-SIP-C1	200	94.8	SG-positive cells	HeLa; peptide pretreat 2 h, then 50 µM sorafenib 2 h
	TAT-SIP-C2		93.1		
	SIP-U1-Antp		94.2		
Dong et al. (2024)	PT-129	5	97.5	SG number per cell	293T, PT129 3 h, then 300 μM AS 20 min
		10	83		293T, 300 μM AS 30 min, then PT-129 1 h
		10	9		293T, 300 μM AS 30 min, then DMSO 1 h

**TABLE 6:** Study details of G3BP1 agonists.

Study	Compound	Dose (μM)	Percentage (%)	Index	Cell line	Others
Fang et al. (2025)	PAE	25	64	% SG-positive cells	HeLa,1 h	​
		50	96.8		HeLa,1 h	
		200	27.5		16HBE,1 h	
Cho et al. (2024)	Tg	1	88.4		U2OS,2 h	Positive control
	TC6	5	31.9			​
	TC12	5	18.8			
	TC39	5	31.2			
Park et al. (2024)	Tg	1	29.9		NIH3T3,2 h	Positive control
	Tg	1	71.7		U2OS,2 h	Positive control
	TC41	5	23.9		NIH3T3,2 h	—
	TC41	5	25.5		U2OS,2 h	—
Amen et al. (2021)	Arsenite	100	100		HEK293T,1 h	Positive control
	Resveratrol	200	95.9			​
	Piceatannol	100	96.9			
	Pterostilbene	100	83.5			
	Tm-stilbene	100	100			
Li et al. (2025)	ICA	0	8.3		HEK293T,0 h	​
		5	9.4			
		10	9.4			
		20	8.9			
		0	8		HEK293T,6 h	​
		5	11.6			
		10	18.8			
		20	23.1			
		0	13.4		HEK293T,12 h	​
		5	18.8			
		10	24.9			
		20	32.9			
		0	14.8		HEK293T,24 h	​
		5	20.2			
		10	27.1			
		20	53.1			
Zhang et al. (2025)	PB3	0	1.91		HT22,OGD/R 24 h	​
		20	4.57			
		50	7.22			
		100	9.88			

#### 7.3.1 Greater cross-laboratory comparability (high robustness)

“Whether SGs form” is a relatively clear cellular phenotype. Compared with total SG area or SG number per cell, the % SG-positive readout is less susceptible to amplified biases introduced by imaging exposure, threshold settings, segmentation algorithms, differences in cell size, and SG fusion/fragmentation at the single-cell level, making it more suitable for cross-study comparisons.

#### 7.3.2 More sensitive to heterogeneity and better reflects population-level responses

Many perturbations (stressors/drugs) yield a mixed response in which some cells respond strongly while others do not. % positivity directly captures “how many cells enter the SG state,” providing an intuitive measure of the fraction of the population adopting a stress-adaptive phenotype. In contrast, area/number-based metrics can be disproportionately driven by a small subset of cells with extremely large SGs or very high granule counts, reducing interpretability at the population level.

#### 7.3.3 Simpler statistics and reporting; well-suited for dose–response and time-course analyses

% positivity is naturally a binary outcome (SG + vs SG−), facilitating dose–response curves, time-course analyses, and effect-size comparisons across conditions (e.g., risk difference, odds ratio). It is also easier to integrate across biological replicates and supports meta-analytic comparisons.

### 7.4 Drug development: Positive control

Across the SG literature, positive controls are not fully standardized: Amen et al. (2021) and Dong et al. (2024) used sodium arsenite, whereas Park et al. (2024) and Cho et al. (2024) used thapsigargin (Tg) (Tables 5, 6). These two inducers trigger SG assembly through distinct upstream stresses and therefore are not always interchangeable. Sodium arsenite induces oxidative stress and robustly activates the integrated stress response (ISR), typically *via* heme-regulated inhibitor (HRI)-mediated eIF2α phosphorylation, leading to rapid translational arrest and highly reproducible formation of canonical G3BP1/2-positive SGs across many adherent cell lines. In contrast, Tg primarily elicits endoplasmic reticulum (ER) stress by perturbing Ca^2+^ homeostasis, activating PERK–eIF2α signaling; its SG induction can be more cell type–and context-dependent and may be confounded by ER remodeling, Ca^2+^-driven signaling, and broader proteostasis effects.

Given the need for cross-laboratory comparability and robust quality control, we recommend adopting sodium arsenite as the default and unified positive control for SG formation assays. A practical standard condition is 0.25–0.5 mM sodium arsenite for 30–60 min (with brief optimization for each cell line to balance induction strength and viability). Thapsigargin (e.g., 0.5–2 µM for 30–60 min) may be included as an optional orthogonal positive control when the biological question specifically concerns ER stress–linked SGs or when arsenite could mechanistically interfere with redox-active compounds under study.

## 8 Discussion and outlook

In aggregate, the field is poised to transition from identifying SG modulators to developing mechanistically grounded therapeutic candidates. The key unmet need is a predictive model that maps ligand properties and cellular context to SG outcomes, particularly within the NTF2L pocket hub where competition and network effects dominate. Achieving this will likely require (i) standardized phenotyping and controls, (ii) robust target engagement validation in cells, (iii) kinetic/occupancy-aware binding characterization, and (iv) stress- and cell type–stratified benchmarking. With these foundations, natural products and synthetic libraries can be screened and prioritized more systematically, and structure-guided optimization can proceed with improved predictability. Ultimately, establishing community-level assay standards and mechanism-resolving datasets may be as impactful for SG drug discovery as large shared resources have been for genomics—enabling iterative refinement, cross-study comparability, and faster translation from SG biology to therapeutic intervention.

## Glossary

ATF4: Activating transcription factor 4
Caprin1: Cell cycle-associated protein 1
cGAS: Cyclic GMP–AMP synthase
eIF2α: Eukaryotic translation initiation factor 2 alpha
EGCG: Epigallocatechin-3-gallate
FGDF: phenylalanine–glycine–aspartate–phenylalanine
G3BP1: Ras-GAP SH3 domain-binding proteins 1
G3BP2: Ras-GAP SH3 domain-binding proteins 2
GCN2: General control nonderepressible 2
GTEx: Genotype-Tissue Expression
HDGF: Hepatoma-derived growth factor
HRI: Heme-regulated inhibitor kinase
IDR: Intrinsically disordered region
JMJD6: Jumonji domain-containing protein 6
LLPS: Liquid–liquid phase separation
MDM2: Mouse double minute 2 homolog
MLOs: Membraneless organelles
nsP3: Non-structural protein 3
p53: Protein p53
PAE: Perillaldehyde
PD-L1: Programmed death-ligand 1
PERK: PKR-like endoplasmic reticulum kinase
PKR: Protein kinase R (double-stranded RNA-dependent protein kinase)
PROTAC: Proteolysis-targeting chimera
PXXP: Proline–X–X–proline
RanBP2: RAN binding protein 2
RasGAP: Ras GTPase-activating protein 1
RBP(s): RNA-binding protein(s)
RGG: Arginine–glycine–glycine (RGG)–rich region
RIOK1: RIO kinase 1
RRM: RNA recognition motif
SART3: Squamous cell carcinoma antigen recognized by T cells 3
SASP: Senescence-associated secretory phenotype
SGs: Stress granules
SIPs: Stress granule inhibitory peptides
SUMO: Small ubiquitin-like modifier
TCGA: The Cancer Genome Atlas
TRIM25: Tripartite motif-containing protein 25
USP10: Ubiquitin specific peptidase 10
